# Functional Analysis of *Hyaluronidase-like* Genes in Ovarian Development of *Macrobrachium nipponense* and Comparative Evaluation with Other Key Regulatory Genes

**DOI:** 10.3390/ijms262110748

**Published:** 2025-11-05

**Authors:** Zhiming Wang, Hao Dong, Hui Qiao, Wenyi Zhang, Shubo Jin, Yiwei Xiong, Zhenghao Ye, Yan Gong, Sufei Jiang, Hongtuo Fu

**Affiliations:** 1 Key Laboratory of Freshwater Aquatic Genetic Resources, Ministry of Agriculture and Rural Affairs, Shanghai Ocean University, Shanghai 201306, China; 2 Key Laboratory of Freshwater Fisheries and Germplasm Resources Utilization, Ministry of Agriculture and Rural Affairs, Freshwater Fisheries Research Center, Chinese Academy of Fishery Sciences, Wuxi 214081, China; 3 Key Laboratory of Mariculture & Stock Enhancement in North China’s Sea, Ministry of Agriculture and Rural Affairs, Dalian Ocean University, Dalian 116023, China; 4 Wuxi Fisheries College, Nanjing Agricultural University, Wuxi 214081, China

**Keywords:** *Macrobrachium nipponense*, *Hyaluronidase-like*, ovarian maturation, RNAi

## Abstract

This study conducted a bioinformatic analysis of two *Hyaluronidase-like* isoforms (*Mn-HyaL1* and *Mn-HyaL2*) in *Macrobrachium nipponense* and investigated their phylogenetic relationships. The open reading frames of *Mn-HyaL1* and *Mn-HyaL2* were 1101 bp (encoding 366 amino acids) and 1164 bp (encoding 387 amino acids), respectively. Both isoforms exhibited similar conserved domains, with an amino acid sequence similarity of 60.21%. Quantitative PCR analysis revealed that the expression levels of *Mn-HyaL1* and *Mn-HyaL2* increased during the mid-to-late phase of each developmental stage, were higher during the reproductive season than in the non-reproductive season, and were more abundant in the hepatopancreas than in other tissues. RNA interference experiments targeting both genes simultaneously demonstrated that knockdown of *Mn-HyaL2* significantly accelerated ovarian development in *M. nipponense*, indicating that *Mn-HyaL* genes function as negative regulators of ovarian maturation. A comparative analysis of multiple genes revealed the following descending order of potency in promoting ovarian development in *M. nipponense*: *Mn-Cholesterol 7-desaturase* > *Mn-Cathepsin L1*. The order of potency in inhibiting ovarian development in *M. nipponense*, from strongest to weakest, was determined to be *Mn-Gonad-inhibiting hormone* > *Mn-HyaL2*.

## 1. Introduction

*Macrobrachium nipponense* is one of the most economically important freshwater aquaculture species in China, with substantial production and significant economic value across East and Southeast Asia. The rapid development of female *M. nipponense* during the breeding season results in the co-occurrence of individuals at vastly different developmental stages under aquaculture conditions. This heterogeneity within populations leads to significant production challenges, including reduced growth rates, low survival rates, and decreased disease resistance due to high-density crowding [1,2]. Therefore, investigating the underlying molecular mechanisms of precocious sexual maturation in *M. nipponense* is of great significance for resolving these production challenges.

The rapid ovarian development process is characterized by an intensive period of protein synthesis [3,4]. Consistent with this, our study observed a marked increase in protein content in the ovary concomitant with a decrease in the hepatopancreas throughout ovarian maturation. This pattern of nutrient redistribution has been well-documented in various crustacean species, including *Fenneropenaeus merguiensis* [5] and *Portunus trituberculatus* [6], supporting the hypothesis that proteins or their precursors are transported from the hepatopancreas to the developing ovary via the hemolymph. Therefore, in our preliminary experiments, we conducted a comparative transcriptomic analysis of hepatopancreas samples from female *M. nipponense* across ovarian stages I to V. The comparison between stage I and stage II revealed that the expression levels of two homologous *Hyaluronidase-like* genes (*Mn-HyaL1* and *Mn-HyaL2*) were significantly upregulated from ovarian stage I to stage II [7].The gene is annotated to the glycosaminoglycan degradation pathway, a component of the lysosomal pathway. Existing evidence has established that this pathway is critically involved in regulating ovarian maturation in *M. nipponense* [8]. Previous studies have established that several genes within this pathway play important roles in regulating ovarian development in *Macrobrachium nipponense* [1,9,10,11]. These results led us to hypothesize that *Mn-HyaL1* and *Mn-HyaL2* could be functionally implicated in the ovarian development of *M. nipponense*, which became the focus of the present investigation.

Based on functional studies published by previous researchers, several genes have been confirmed to regulate ovarian development in *M. nipponense* (Table 1). A major limitation, however, lies in the heterogeneity of experimental settings—such as season, tank size, and measured parameters—which complicates cross-study comparisons. To address this gap, a controlled comparative analysis is needed to rank these genes by efficacy, information vital for aquaculture productivity. Based on an analysis of published functional studies, key regulatory genes of ovarian development in *Macrobrachium nipponense* were selected for comparative RNA interference (RNAi) assays. Among these, *Cholesterol 7-desaturase* (*Mn-CH7D*) [2] and *Mn-Cathepsin L1* (*Mn-CTSL1*) [10] were identified as strong promoters of ovarian development, consistently inducing an advance of more than two developmental stages in RNAi-silencing experiments, while *Mn-Gonad-inhibiting hormone* (*Mn-GIH*) [12] acted as a potent suppressor. These well-characterized regulators were therefore prioritized for further functional comparison under identical long-term RNAi conditions.

In this study, bioinformatic analyses were conducted to characterize the sequences and phylogenetic relationships of *Mn-HyaL1* and *Mn-HyaL2*. The expression patterns of these two genes were examined by qPCR across various developmental stages, sexes, seasons, and tissues, with a particular focus on their expression during ovarian and hepatopancreas development. Furthermore, RNA interference (RNAi) was employed to knock down *Mn-HyaL1*, *Mn-HyaL2*, and other previously validated key regulators of ovarian development in *M. nipponense* in parallel experimental groups. This approach enabled us not only to investigate the regulatory functions of these genes in ovarian maturation but also to compare their relative functional strengths. The findings of this study may provide valuable insights for addressing the issue of precocious ovarian maturation in female *M. nipponense*.

## 2. Results

### 2.1. Characterization of the Full-Length Sequences of Mn-HyaL1 and Mn-HyaL2

**Figure 1:**
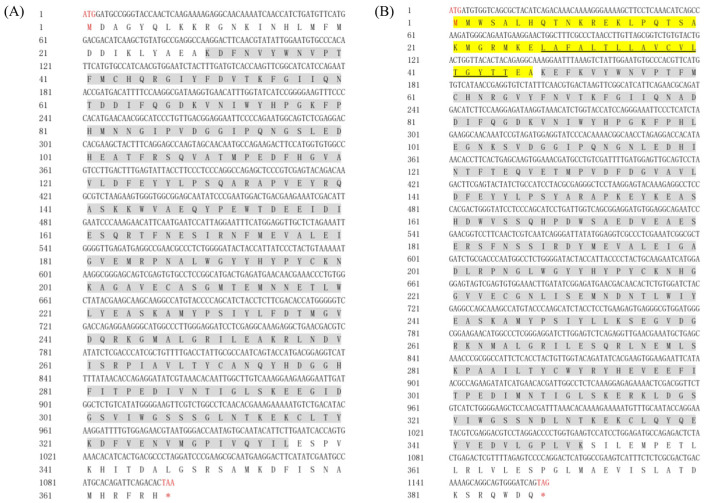
Nucleotide and deduced amino acid sequences of (**A**) *Mn-HyaL1* and (**B**) *Mn-HyaL2*. The start (ATG) and stop codons (TAA/TAG/TGA) are highlighted in red. The termination codon in the amino acid sequence is indicated by an asterisk (*). Yellow-shaded regions represent signal peptide sequences, while gray-shaded areas denote conserved functional domains of the genes. The black solid line marks the transmembrane domain of the gene.

The open reading frame (ORF) of the *hyaluronidase-like1* gene in *Macrobrachium nipponense* was 1101 bp in length, encoding a protein of 366 amino acids, and it was designated as *Mn-HyaL1* (GenBank accession no. PV941746). The ORF of the *hyaluronidase-like2* gene in *M. nipponense* was 1164 bp, encoding a protein of 387 amino acids, and was designated as *Mn-HyaL2* (GenBank accession no. PV941747). The cDNA and deduced amino acid sequences of both *Mn-HyaL1* and *Mn-HyaL2* are presented in Figure 1.

Bioinformatic analysis revealed that the molecular weight (Mw) of the *Mn-HyaL1* protein is 41,906.46 Da, with a theoretical isoelectric point (pI) of 5.41. Analysis of the full-length amino acid composition indicated that glycine (G) was the most abundant residue, constituting 7.4% of the sequence. The protein contains 36 positively charged residues (Arg + Lys) and 49 negatively charged residues (Asp + Glu). The predicted molecular formula is C_1877_H_2841_N_497_O_554_S_21_. Secondary structure prediction showed that *Mn-HyaL1* comprises 9 α-helices, 7 η-helices, 9 β-sheets, and 3 β-turns; the sequential arrangement of these elements is provided in Figure S1. Furthermore, *Mn-HyaL1* was predicted to lack both a signal peptide and transmembrane domains.

Bioinformatic analysis indicated that the *Mn-HyaL2* protein has a molecular weight (Mw) of 44,331.21 Da and a theoretical isoelectric point (pI) of 4.99. Analysis of its full-length amino acid composition showed that leucine (L) was the most abundant residue at 9.3%. The protein contains 37 positively charged residues (Arg + Lys) and 56 negatively charged residues (Asp + Glu), with a predicted molecular formula of C_1986_H_3031_N_515_O_598_S_20_. The predicted secondary structure consists of 9 α-helices, 5 η-helices, 10 β-sheets, and 5 β-turns; the sequential arrangement is detailed in Figure S1. *Mn-HyaL2* was predicted to contain one transmembrane domain located between residues 28 and 45. Additionally, a signal peptide (Sec/SPI type) was identified at the N-terminus (residues 1–47), with a cleavage site between residues 47 and 48. The signal peptide sequence was determined to be MMWSALHQTNKREKLPQTSAKMGRMKELAFALTLLAVCVLTGYTTEA. The region from residue 48 to 387 was predicted to constitute the non-cytoplasmic domain.

Sequence analysis revealed that both *Mn-HyaL1* and *Mn-HyaL2* contain the same conserved domain, Hyaluronidase (Accession: pfam01630), located at residues 30–336 in *Mn-HyaL1* and residues 48–351 in *Mn-HyaL2*. Furthermore, four identical functional domains were identified in both proteins: Hyaluronidase (IPR018155), Aldolase-type TIM barrel (IPR013785), Glycoside hydrolase superfamily (IPR017853), and Glycoside hydrolase family 56, bee venom hyaluronidase (IPR001329).

### 2.2. Analysis of Species Similarity and Phylogeny of Mn-HyaL1 and Mn-HyaL2

Sequence alignment of *Mn-HyaL1* and *Mn-HyaL2* was performed using DNAMAN 9.0, revealing a similarity of 60.21% between the two isoforms. Multiple sequence alignment was then conducted using *Mn-HyaL1* and *Mn-HyaL2* alongside homologous genes from other species, which included, in order: *Macrobrachium rosenbergii*, *Palaemon carinicauda*, *Halocaridina rubra*, *Cherax quadricarinatus*, *Penaeus indicus*, *Penaeus chinensis*, and *Penaeus vannamei*. Nucleotide sequence similarities were calculated using the p-distance method. The results showed that *Mn-HyaL1* shared sequence similarities of 93.56%, 63.77%, 50.99%, 46.93%, 46.58%, 45.35%, and 44.86% with the corresponding genes from these species, respectively. Similarly, *Mn-HyaL2* exhibited similarities of 61.78%, 79.01%, 54.98%, 46.79%, 48.84%, 46.18%, and 47.58%, respectively (Figure S2).

**Figure 2:**
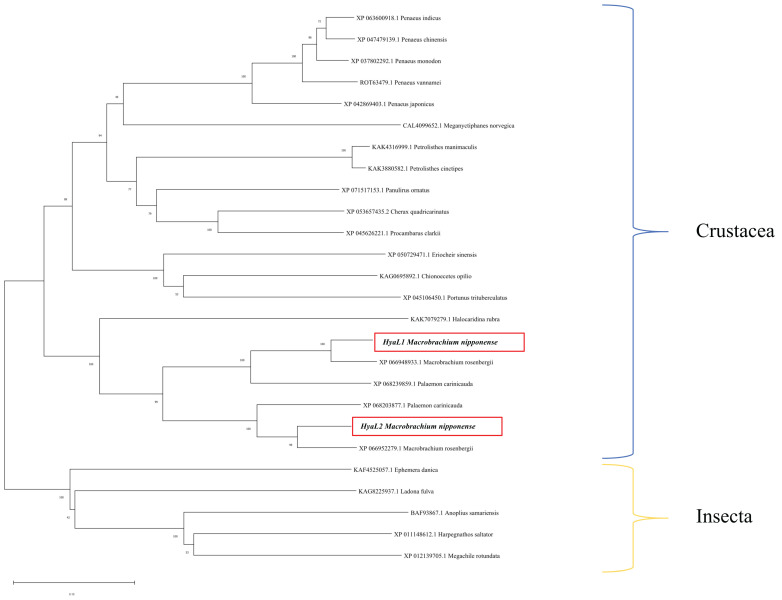
Phylogenetic tree analysis of *Mn-HyaL1* and *Mn-HyaL2*. The numbers on the branch represent the bootstrap percentages of the phylogenetic tree. Bootstrap copy to 1000. The terminal numbers correspond to GenBank accession numbers. The target genes are marked with the red boxes. The evolutionary history was inferred using the Neighbor-Joining method.

A phylogenetic tree was constructed using MEGA 11.0 based on the amino acid sequences of *Mn-HyaL1*, *Mn-HyaL2*, and their homologs from other species. The analysis revealed that *Mn-HyaL1* and *Mn-HyaL2* did not cluster directly with each other. Instead, each first clustered with distinct homologs from *Macrobrachium rosenbergii* and *Palaemon carinicauda* (both belonging to the family Palaemonidae), before subsequently grouping together. This combined cluster then grouped with other crustaceans, such as *Halocaridina rubra*, and finally with insects (Figure 2).

### 2.3. Analysis of Spatiotemporal Expression Patterns of Mn-HyaL1 and Mn-HyaL2

#### 2.3.1. Analysis of Stage-Specific Expression Patterns of *Mn-HyaL1* and *Mn-HyaL2*

**Figure 3:**
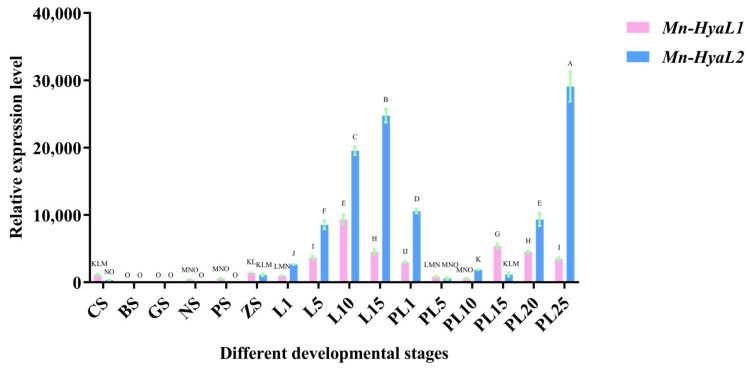
Quantitative analysis of *Mn-HyaL1* and *Mn-HyaL2* expression patterns during various developmental stages by qPCR. Data are presented as mean ± SD (n = 6). Different letters above bars indicate significant differences according to the Compact Letter Display (CLD) system (*p* < 0.05). Developmental stages from CS to PL25 are detailed in Table S1. CS: cleavage stage; BS: blastula stage; GS: gastrula stage; NS: nauplius stage; PS: protozoea stage; ZS: zoea stage; L1–L15: days post hatching (larval stage); PL1–PL25: days post metamorphosis (postlarval stage).

The expression patterns of *Mn-HyaL1* and *Mn-HyaL2* during embryonic and post-embryonic developmental stages are shown in Figure 3. During early embryonic development (from CS to ZS), *Mn-HyaL1* expression remained consistently high at both the Cleavage stage (CS) and Zoea Stage (ZS) with no significant difference between these two stages, whereas *Mn-HyaL2* expression peaked specifically at ZS. During the post-embryonic developmental stage (from L1 to L15), *Mn-HyaL1* expression was highest at the L10 stage, while *Mn-HyaL2* expression peaked at the L15 stage. In the subsequent stage (from PL1 to PL25), *Mn-HyaL1* expression reached its maximum at the PL15 stage, whereas *Mn-HyaL2* showed the highest expression at the PL25 stage. Overall, the highest expression level of *Mn-HyaL1* was observed at the L10 stage, and that of *Mn-HyaL2* was observed at the PL25 stage.

#### 2.3.2. Analysis of Tissue-Specific Expression Patterns of *Mn-HyaL1* and *Mn-HyaL2*

**Figure 4:**
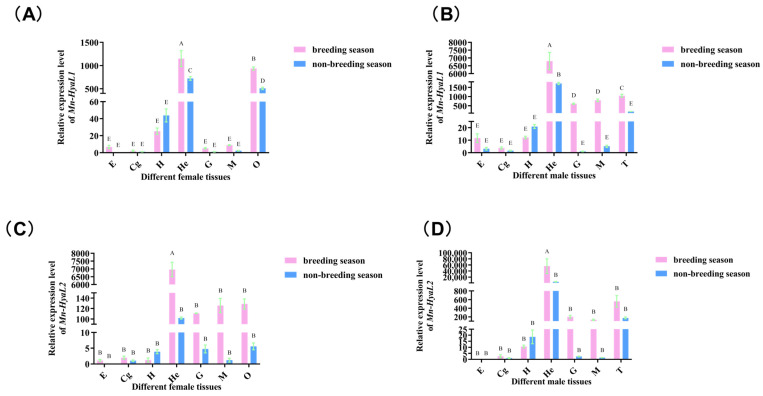
Tissue expression analysis of (**A**) *Mn-HyaL1* in females, (**B**) *Mn-HyaL1* in males, (**C**) *Mn-HyaL2* in females, and (**D**) *Mn-HyaL2* in males during breeding season and non-breeding season periods quantified by qPCR. E: eyestalk; Cg: cerebral ganglion; H: heart; He: hepatopancreas; G: gill; M: muscle; O: ovary; T: testis. Data are presented as mean ± SD (n = 6). Different letters above bars indicate significant differences according to the Compact Letter Display (CLD) system (*p* < 0.05).

As shown in Figure 4, the highest expression levels of both *Mn-HyaL1* and *Mn-HyaL2* were detected in the hepatopancreas, followed by the gonads. Furthermore, in most tissues examined, the expression of these genes was generally higher during the reproductive season than during the non-reproductive season, with this pattern being observed in both female and male shrimp.

#### 2.3.3. Analysis of Stage-Specific Expression Patterns of *Mn-HyaL1* and *Mn-HyaL2* in Hepatopancreatic and Ovarian Tissues

**Figure 5:**
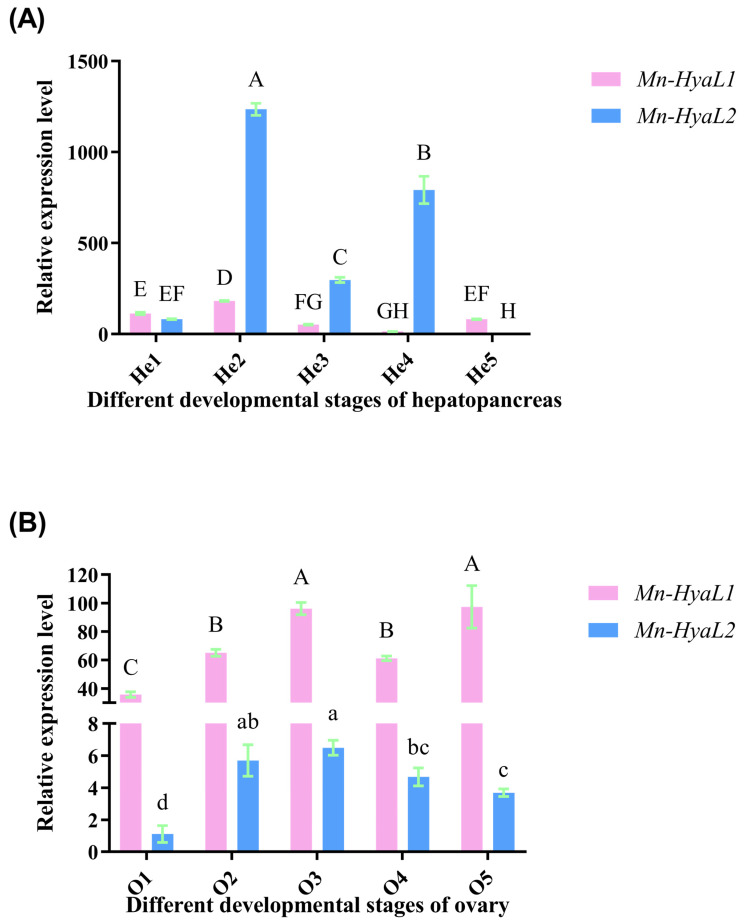
qPCR analysis of *Mn-HyaL1* and *Mn-HyaL2* expression patterns during distinct developmental stages in (**A**) hepatopancreatic and (**B**) ovarian tissues. Data are presented as mean ± SD (n = 6). Different letters above bars indicate significant differences according to the Compact Letter Display (CLD) system (*p* < 0.05). Different superscript letters indicate statistically significant differences based on the combined analysis of *Mn-HyaL1* and *Mn-HyaL2* expression data in (**A**) (*p* < 0.05). Significant differential expression was observed between *Mn-HyaL1* and *Mn-HyaL2* across all developmental stages in ovarian tissues (*p* < 0.01). In (**B**), uppercase letters indicate statistically significant differences among *Mn-HyaL1* expression groups (*p* < 0.05), whereas lowercase letters denote significant variations among *Mn-HyaL2* expression groups (*p* < 0.05). The developmental stages from O1–O5 and He1–He5 are defined in Table S1. O1: oocyte stage; O2: primary vitellogenesis stage; O3: secondary vitellogenesis stage; O4: ripe stage; O5: emptying stage. He1-He5: hepatopancreas corresponding to O1–O5 at the same stage.

In the hepatopancreas (Figure 5A), the expression levels of both *Mn-HyaL1* and *Mn-HyaL2* increased significantly from stage He1 to He2 (*p* < 0.01), peaking at stage He2. The expression at He2 was significantly higher than that at all other stages (*p* < 0.01). The lowest expression level of *Mn-HyaL1* was observed at stage He4, whereas *Mn-HyaL2* exhibited its minimum expression at stage He5.

In the ovaries (Figure 5B), the expression levels of both *Mn-HyaL1* and *Mn-HyaL2* increased significantly from stage O1 to O2 (*p* < 0.01). The highest expression of *Mn-HyaL1* was observed at stage O5, while *Mn-HyaL2* peaked at stage O3. The lowest expression for both genes occurred at stage O1, which was significantly lower than that at all other stages (*p* < 0.01). Additionally, the expression level of *Mn-HyaL1* was significantly higher than that of *Mn-HyaL2* at each ovarian stage (*p* < 0.05).

### 2.4. Functional Characterization and Comparative Analysis of Genes

#### 2.4.1. Evaluation of RNA Interference Efficiency

**Figure 6:**
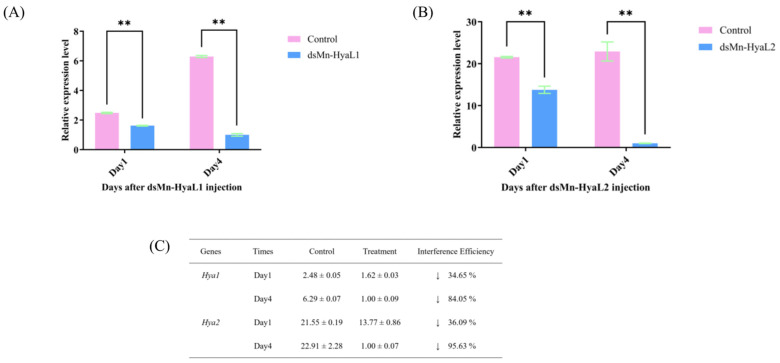
The expression levels of (**A**) *Mn-HyaL1* and (**B**) *Mn-HyaL2* in the hepatopancreas of *Macrobrachium nipponense* after dsRNA injection, with (**C**) corresponding interference test data. Values represent mean ± SD (n = 6). Asterisks indicate statistical significance: ** (*p* < 0.01). Downward arrows (↓) denote expression suppression.

To further investigate the functional roles of *Mn-HyaL1* and *Mn-HyaL2* in ovarian maturation, RNA interference (RNAi) was employed. As shown in Figure 6, the knockdown efficiencies of dsMn-HyaL1 were 34.65% and 84.05% on day 1 and day 4 post-injection, respectively (*p* < 0.01). Similarly, dsMn-HyaL2 achieved knockdown efficiencies of 36.09% and 95.63% on day 1 and day 4, respectively (*p* < 0.01). Both dsRNAs induced significant gene silencing, with dsMn-HyaL2 exhibiting a higher knockdown efficiency. For *Mn-CH7D* [2], *Mn-CTSL1* [10], and *Mn-GIH* [12], we employed the identical dsRNA sequences and experimental conditions that were previously validated in their original functional studies. These studies reported knockdown efficiencies of 89.92% for Mn-CH7D, 99.79% for Mn-CTSL1, and 83% for Mn-GIH. By maintaining complete consistency with these established protocols, we ensured comparable silencing efficacy across all target genes in the present comparative analysis.

#### 2.4.2. Comparative Functional Analysis of Key Genes in Ovarian Development of *M. nipponense*

**Figure 7:**
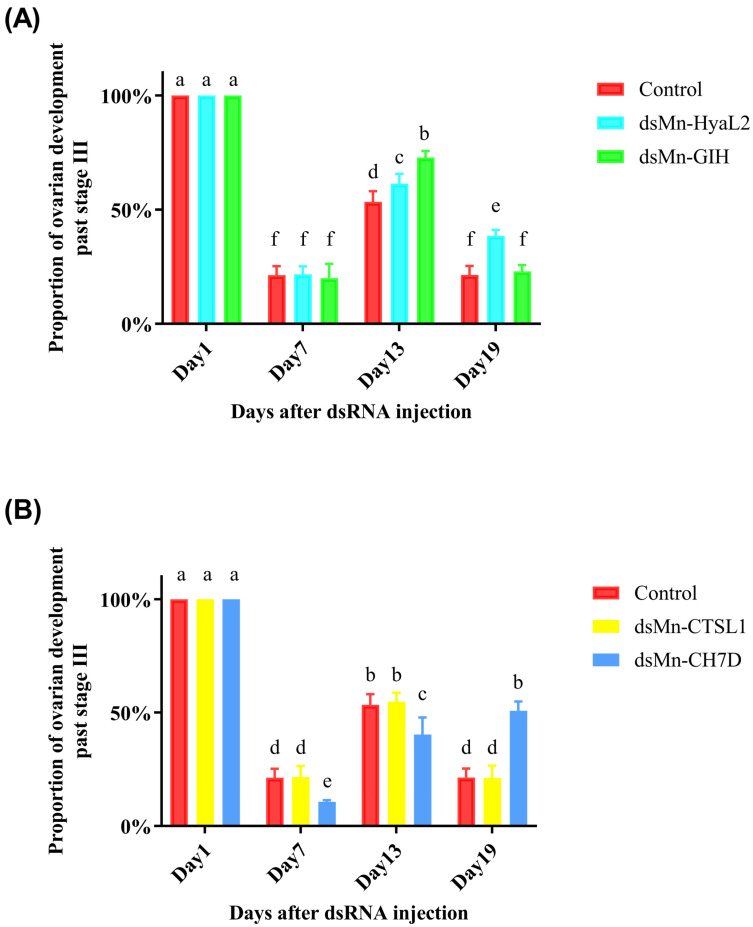
Temporal changes in the percentage of female *Macrobrachium nipponense* exhibiting ovarian development beyond stage III after dsRNA injection. (**A**) Genes inhibiting ovarian development. (**B**) Genes promoting ovarian development. Data represent the mean ± SD from three independent experiments. Different letters above bars indicate significant differences according to the Compact Letter Display (CLD) system (*p* < 0.05).

A comparison of ovarian development between the experimental and control groups is presented in Figure 7. At the start of the experiment, all shrimp were at stage O3. By day 6, all groups had entered a new reproductive cycle. From day 6 to day 12, the proportion of shrimp with ovaries beyond stage III gradually increased in all groups. By day 18, all groups except the *Mn-CH7D* group had entered a third reproductive cycle. Overall, the order of ovarian development speed from slowest to fastest following RNAi was: *Mn-CH7D* experimental group < *Mn-CTSL1* experimental group = control group < *Mn-HyaL2* experimental group < *Mn-GIH* experimental group. Due to a high mortality rate, the *Mn-HyaL1* experimental group was excluded from the statistical analysis as the resulting data were insufficient for meaningful interpretation.

## 3. Discussion

While hyaluronidases are well-characterized in vertebrate and microbial systems for their roles in extracellular matrix remodeling [28,29], their functions in crustacean reproduction remain largely unexplored [30]. The present study provides the first functional characterization of two *hyaluronidase-like* genes, *Mn-HyaL1* and *Mn-HyaL2*, in the ovarian development of *Macrobrachium nipponense*.

The *HyaL* enzymes characterized in this study are categorized as endo-hyaluronidases (hyaluronoglucosaminidase, EC 3.2.1.35) [31], a class of glycoside hydrolases predominantly sourced from vertebrates and venoms, which degrade hyaluronic acid via hydrolysis of β-1,4-glycosidic bonds to produce specific oligosaccharides and can also target chondroitin sulfate or dermatan sulfate [32], thus differing fundamentally from other hyaluronidase types (EC 3.2.1.36; EC 4.2.2.1) [33,34,35].

Evidence from reproductive studies suggests conserved roles for hyaluronidases in fertilization processes, as exemplified by the functional similarity between bee venom hyaluronidase and the mammalian sperm membrane protein PH-20, both facilitating gamete interaction through hyaluronic acid degradation [36,37,38]. Hyaluronidase activity is significantly correlated with fertilization rates [38,39,40]. In ovarian contexts, hyaluronidase activity has been linked to follicular atresia and tissue remodeling [28,41]. Furthermore, hyaluronidase dysregulation has been associated with several ovarian disorders [42,43,44]. An age-related decline in hyaluronic acid content is observed in the ovarian stroma [45]. But its specific role in crustacean ovarian development remains largely uncharacterized. Limited studies have revealed that hyaluronidase activity in hepatopancreas homogenates of the red king crab (*Paralithodes camtschaticus*) is an order of magnitude higher than that of commercial preparations, suggesting its potential as a high-quality source for hyaluronidase applications in cosmetics and medicine [46,47]. The present study addresses this gap by functionally characterizing two *hyaluronidase-like* genes in *Macrobrachium nipponense*, providing the first insights into their regulatory contributions to ovarian maturation in crustaceans.

Bioinformatic analysis revealed that both genes belong to the Glycoside Hydrolase Family 56 (GH56), a group typically associated with arthropod venoms where they act as “spreading factors” by degrading extracellular matrix components [48,49,50,51]. Notably, sequence analysis indicated structural divergence between the two isoforms: *Mn-HyaL2* possesses an N-terminal signal peptide and transmembrane domain absent in *Mn-HyaL1*, suggesting distinct subcellular localization and potential functional specialization.

The screening results of the *Mn-HyaL1* and *Mn-HyaL2* genes were obtained from a preliminary experiment. In the present study, *Mn-HyaL1* and *Mn-HyaL2* were identified from a comparative transcriptomic analysis of the hepatopancreas of *M. nipponense* at ovarian stages O1 and O2, where their expression increased significantly from stage O1 to O2 [7]. Subsequent KEGG enrichment analysis revealed that both genes are involved in the “Lysosome” signaling pathway (KEGG: map04142), which has been previously demonstrated to be closely associated with ovarian development in *M. nipponense* [8]. The relationship between this pathway and ovarian development, as well as other regulatory genes within the pathway, has been discussed in earlier studies [9].

The observed involvement of *Mn-HyaL1* and *Mn-HyaL2* in ovarian development can be understood within the functional framework of lysosomal activity. In crustaceans, lysosomes contribute to steroidogenesis through two well-established mechanisms: facilitating steroid precursor availability and modulating regulators of steroid production, thereby maintaining endocrine homeostasis [52]. Specifically, lysosomes enable cholesterol release from endocytosed LDL via coordinated action of enzymes and transporters such as *NPC1* and *NPC2* [53], while also participating in the degradation of key regulatory complexes including LH-LHR, FSH-FSHR, and PGF2α receptors [54,55,56].

As hyaluronidases, the primary function of *Mn*-*HyaL1* and *Mn*-*HyaL2* lies in hydrolyzing hyaluronic acid—a key component of the extracellular matrix. This functional similarity to lysosomal hydrolases suggests their potential involvement in analogous processes during ovarian maturation. Notably, hyaluronidase activity has been shown to play a key role in the early phases of folliculogenesis by negatively regulating ovarian follicle growth and survival [57]. This regulatory function may operate through modulation of the extracellular matrix environment surrounding developing follicles, thereby influencing follicular development and oocyte maturation.

We hypothesize that *Mn-HyaL1* and *Mn-HyaL2* may facilitate follicular remodeling, nutrient mobilization, and hormonal regulation through the controlled degradation of hyaluronan-rich matrices. Such activity could potentially influence the availability of signaling molecules or growth factors embedded in the extracellular matrix, thereby indirectly supporting the lysosome-mediated pathways essential for ovarian development. The distinct expression patterns and functional characteristics of these two isoforms suggest they may fulfill complementary roles at different stages of ovarian maturation. Further studies are needed to elucidate the specific mechanisms by which these hyaluronidase isoforms contribute to reproductive maturation in *M. nipponense*, particularly their potential interplay with established lysosomal pathways.

Bioinformatic analysis revealed that *Mn-HyaL1* and *Mn-HyaL2* share highly similar conserved and functional domains, suggesting overall functional similarity. However, their nucleotide and amino acid sequence alignment showed limited homology, with an amino acid identity of only 60.21%. Furthermore, *Mn-HyaL2* possesses an N-terminal signal peptide and a transmembrane domain, which are absent in *Mn-HyaL1*. Phylogenetic analysis also indicated that *Mn-HyaL1* and *Mn-HyaL2* do not form a direct cluster. These observations suggest that while the two isoforms may share core functions due to their conserved domain architecture, structural differences could lead to functional distinctions in specific aspects such as subcellular localization or regulatory mechanisms.

The distinct domain architectures of *Mn-HyaL1* and *Mn-HyaL2* provide important insights into their potential functional differentiation. While both proteins share identical catalytic domains (Hyaluronidase pfam01630) and functional motifs, the exclusive presence of an N-terminal signal peptide and transmembrane domain in *Mn-HyaL2* suggests divergent subcellular targeting mechanisms. This structural distinction supports the hypothesis that *Mn-HyaL2* may function as a secreted or membrane-associated protein, potentially modifying extracellular matrix components or participating in cell surface signaling events. In contrast, *Mn-HyaL1*, lacking these targeting signals, likely operates intracellularly, possibly regulating internal pools of hyaluronic acid or other glycosaminoglycans.

This spatial segregation could explain the observed functional specialization between the two isoforms, as they would access distinct substrates and interact with different regulatory partners within separate cellular compartments. The combination of conserved catalytic domains with divergent targeting sequences suggests an evolutionary strategy where core enzymatic function is maintained while cellular localization and substrate specificity are diversified. This pattern aligns with the phylogenetic evidence indicating an ancient gene duplication event followed by functional divergence, ultimately yielding complementary but non-redundant roles in ovarian development regulation.

In the tissue-specific expression analysis, although the expression levels of *Mn-HyaL1* and *Mn-HyaL2* varied across different stages, both isoforms were generally highly expressed during the mid-to-late phases of each stage. These late phases often represent critical periods in the development of *M. nipponense* that demand substantial energy expenditure. Considering the fundamental hydrolytic function of *hyaluronidase-like* genes, we hypothesize that the two *Mn-HyaL* isoforms are involved in energy regulation during these key stages. This notion is further supported by the concurrent upregulation of both isoforms during the reproductive season, a period of heightened energy demands for activities such as growth, mating, and spawning. Additionally, the expression of both *Mn-HyaL1* and *Mn-HyaL2* was significantly higher in the hepatopancreas than in other tissues. As the hepatopancreas is the primary organ supplying energy to the ovaries, and given that both isoforms showed increased expression from stage O1/He1 to O2/He2—a period characterized by rapid vitellogenesis and the transfer of energy substrates like glycoproteins from the hepatopancreas to the ovaries [7,8]—these results collectively reinforce the hypothesis that *Mn-HyaL* isoforms may participate in the energy regulation supporting ovarian development.

In the RNA interference (RNAi) experiment, after 18 days, all experimental groups except the *Mn-CH7D* group had entered the third reproductive cycle, indicating that *Mn-CH7D* has the most significant promotive effect on ovarian development. Conversely, the ovarian development speed in the *Mn-HyaL2* and *Mn-GIH* groups was faster than that in the control group, identifying them as inhibitory genes, with *Mn-GIH* exhibiting a stronger effect than *Mn-HyaL2* (Figure 7A). No significant difference was observed between the *Mn-CTSL1* group and the control group, a finding consistent with previous reports on *Mn-CTSL2* [11]. The *Mn-HyaL1* experimental group exhibited an unexpectedly high mortality rate, which precluded meaningful data analysis. This suggests that *Mn-HyaL1* may play a critical role in essential physiological processes such as immunity, molting, or other vital growth pathways. This intriguing phenomenon will be the focus of our future investigations. Future studies could explore alternative interference methods (e.g., oral delivery of dsRNA) to mitigate the high mortality observed in this study [58].

The relationship between *Mn-HyaL1* and *Mn-HyaL2* genes is a key point to be discussed. The evolutionary relationship between *Mn-HyaL1* and *Mn-HyaL2* provides important context for interpreting their functional roles. Phylogenetic analysis revealed that *Mn-HyaL1* and *Mn-HyaL2* did not form a distinct clade, with each instead clustering more closely with its putative ortholog from *Macrobrachium rosenbergii* (Figure 2). This topology suggests that the divergence of these two genes is an ancient event, likely predating the speciation of *M. nipponense* and *M. rosenbergii*, and that they are therefore best classified as orthologs rather than recently duplicated paralogs. However, despite this orthologous origin, several lines of evidence indicate that *Mn-HyaL1* and *Mn-HyaL2* have undergone significant functional divergence within *M. nipponense*. Their relatively low amino acid sequence similarity (60.21%), distinct spatiotemporal expression profiles across all examined tissues and developmental stages, and the markedly different phenotypic consequences of their RNAi knockdown (e.g., the high mortality observed following *Mn-HyaL1* silencing) collectively support this notion of functional specialization. Thus, while *Mn-HyaL1* and *Mn-HyaL2* share a common ancestral gene, evolutionary pressures appear to have shaped them into regulators with non-redundant, and potentially complementary, functions in ovarian development.

In summary, this study successfully identified the following key findings and conclusions: (1) The full-length cDNA sequences of *Mn-HyaL1* and *Mn-HyaL2* were cloned and characterized for the first time. (2) The spatiotemporal expression patterns of *Mn-HyaL1* and *Mn-HyaL2* were systematically investigated across different developmental stages, sexes, seasons, and tissues, with a particular focus on their expression during ovarian and hepatopancreas development. (3) Functional validation via RNAi confirmed that *Mn-HyaL* acts as a negative regulator of ovarian development in *M. nipponense*. This finding is significant as most previously identified regulators in this species are positive regulators. Notably, this study provides the first evidence of a regulatory role for a *Hyaluronidase-like* gene in ovarian development, and represents the first functional characterization of a *HyaL* gene in crustaceans. (4) The relative potency of the studied genes in promoting ovarian development was determined to be *Mn-CH7D* > *Mn-CTSL1*. The inhibitory potency on ovarian development followed the order: *Mn-GIH* > *Mn-HyaL2*. This study is the first to conduct a direct comparative analysis of these key regulators under identical conditions, thereby identifying the most potent positive and negative regulators. These results contribute to elucidating the molecular mechanisms underlying ovarian development in *M. nipponense* and have important implications for the genetic breeding of improved germplasm resources in aquaculture.

## 4. Materials and Methods

### 4.1. Experimental Animals and Breeding Conditions

Healthy female specimens of *Macrobrachium nipponense* used in this study were obtained from the Freshwater Fisheries Research Center of the Chinese Academy of Fishery Sciences in Wuxi City, Jiangsu Province, China. All selected shrimp were at ovarian stage III and had a uniform body weight (1 ± 0.18 g). The shrimp were reared in indoor recirculating aquaculture systems using fiberglass tanks (95 cm × 45 cm × 45 cm). The tanks were equipped with plastic aquatic plants to provide shelter. The shrimp were fed twice daily (morning and evening) with a commercial diet at a rate of approximately 5% of their total body weight per feeding. The water temperature was maintained at an average of 29 °C.

### 4.2. Tissue Sample Collection

Various tissues, including the eye, cerebral ganglion, heart, hepatopancreas, gill, muscle, and gonad, were rapidly dissected from *M. nipponense* and immediately frozen in liquid nitrogen. All collected samples were subsequently stored at −80 °C until further analysis. Additionally, individual shrimp samples at different developmental stages, ranging from embryonic to larval phases, were also collected and similarly stored at −80 °C for subsequent experiments. The specific criteria for classifying ovarian stages and embryonic developmental stages are provided in Table S1 [9,59].

*M. nipponense* specimens were euthanized by rapid freezing using liquid nitrogen. Live prawns were transferred to a sealed container filled with an adequate volume of liquid nitrogen, where the ultra-low temperature (−196 °C) induced instantaneous loss of consciousness and rapid death. This method minimizes potential suffering and meets ethical requirements for crustacean euthanasia. Death was confirmed by the absence of movement and rigidification of the body.

### 4.3. Molecular Biology Methods

Total RNA was extracted from *M. nipponense* tissues at various developmental stages using RNAiso Easy reagent (Takara, Dalian, China). Tissue samples were homogenized by a combination of manual grinding (for larger tissues) and mechanical disruption using a tissue homogenizer to ensure complete cell lysis. For early developmental stages where tissue mass was limited, multiple individuals were pooled to obtain sufficient RNA yield per sample. RNA integrity was assessed by 1.2% agarose gel electrophoresis. First-strand cDNA was synthesized from the RNA templates using the M-MLV Reverse Transcriptase kit (Takara, Kusatsu, Japan) and stored at −20 °C for subsequent quantitative real-time PCR (qPCR) analysis to examine the expression patterns of *Hyaluronidase-like* genes. The elongation factor gene (*EIF*), which has been validated in previous studies [60], was used as the internal reference. Gene expression levels were calculated using the 2^−ΔΔCT^ method [61].

### 4.4. Bioinformatics Analysis

The cDNA fragments of the target genes, *Mn-HyaL1* and *Mn-HyaL2*, were obtained from a cDNA library derived from the hepatopancreas of *M. nipponense* at different ovarian stages, which was constructed in our laboratory during previous research [7]. The tools and websites used for analyzing the nucleotide and amino acid sequences of the target genes are listed in Table S2. All primers used in this study are provided in Table S3.

### 4.5. RNA Interference Experiment

Double-stranded RNA (dsRNA) was synthesized using the Transcript Aid™ T7 High Yield Transcription Kit (Fermentas, Inc., Waltham, MA, USA), with primers listed in Table S3. The concentration of the synthesized dsRNA was measured at 260 nm using a biophotometer (Eppendorf, Hamburg, Germany).

For the short-term interference assay, hepatopancreas samples were collected on days 1 and 4 after dsRNA injection for cDNA synthesis. The knockdown efficiency was determined by quantifying the relative expression levels of the target genes in the dsMn-HyaL1 and dsMn-HyaL2 groups via qPCR. The dsRNA yielding the higher interference efficiency was selected for the long-term experiment.

In the long-term trial, a total of 720 healthy female *M. nipponense* at ovarian stage III were randomly and equally distributed into 18 aquaculture tanks. These represented five experimental groups: *Mn-HyaL1*, *Mn-HyaL2*, *Mn-CH7D*, *Mn-CTSL1*, *Mn-GIH*, and a control group, each with three replicates. Shrimp in the treatment groups received an intracardiac injection of the corresponding dsRNA at a dose of 4 μg per gram of body weight [2], while the control group was injected with an equivalent amount of dsGFP. Booster injections were administered every 6 days. The ovarian development stage of each shrimp were recorded daily. The percentage of shrimp exhibiting ovarian development beyond stage III was calculated.

### 4.6. Statistical Analysis

All quantitative data are presented as the mean ± standard deviation (SD). Data analysis was performed using SPSS Statistics 24.0. One-way analysis of variance (ANOVA) followed by Duncan’s test was used for multiple comparisons to assess significant differences between the control and treatment groups. A significance level of *p* < 0.05 was applied. Relative gene expression was calculated using the 2^−ΔΔCT^ method.

## 5. Conclusions

In conclusion, this study provides the first comprehensive functional characterization of *Mn-HyaL1* and *Mn-HyaL2* in a crustacean species through an integrated approach combining bioinformatics, molecular, and physiological analyses. Notably, we demonstrate for the first time in crustaceans that *Mn-HyaL* functions as a negative regulator of ovarian development in *Macrobrachium nipponense*, which also represents the initial discovery of a regulatory role for a *Hyaluronidase-like* gene in the ovarian development process. Furthermore, this work is the first to conduct a direct comparative analysis of key regulatory genes under identical conditions in *M. nipponense*, identifying the most potent positive and negative regulators. These findings significantly contribute to elucidating the molecular mechanisms governing ovarian development in this commercially important species and hold substantial promise for the genetic improvement of germplasm resources in aquaculture.

## Figures and Tables

**Table 1:** Research Advances regarding Regulatory Genes Associated with Ovarian Development in *Macrobrachium nipponense*.

First Author	Genes	Methodological Approach	Year	Reference
Jisheng Wang	*Cholesterol 7-desaturase*	The proportion of ovarian development over the third stage and gonadosomatic index (GSI)	2023	[2]
Jisheng Wang	*Juvenile hormone epoxide hydrolase*	The proportion of ovarian development over the third stage and GSI	2024	[13]
Yalu Zheng	*CYP302A1*	The proportion of ovarian development over the third stage and GSI	2023	[14]
Sufei Jiang	*NPC intracellular cholesterol transporter 1*	The proportion of ovarian development over the third stage and GSI	2024	[15]
Sufei Jiang	*CYP315A1*	The proportion of ovarian development over the third stage and GSI	2025	[16]
Mengying Zhang	*Methyl farnesoate epoxidase*	The proportion of ovarian development over the third stage and GSI	2024	[17]
Hongkun Bai	*Vitellogenin* *(Vg)*	Ovarian developmental stages, relative expression levels of *Vg* and GSI	2015	[18]
Junpeng Zhu	*Cathepsin L1*	Ovarian developmental stages, relative expression levels of *Vg* and GSI	2021	[10]
Zhenyu Zhou	*Cyclin A*	Ovarian developmental stages and GSI	2021	[19]
Dan Cheng	*Cathepsin D*	Ovarian developmental stages and GSI	2022	[1]
Hui Qiao	*Gonad-inhibiting hormone*	Ovarian developmental stages and GSI	2015	[12]
Hongkun Bai	*Vitellogenin receptor*	Relative expression levels of *Vg* and GSI	2016	[20]
Sufei Jiang	*Cathepsin L2*	Relative expression levels of *Vg* and GSI	2022	[11]
Zhiming Wang	*β-Hexosaminidase A*	The proportion of ovarian development over the third stage	2025	[9]
Tianyong Chen	*XRN1*	The proportion of ovarian development over the third stage	2023	[21]
Wenshan Cui	*Ribosomal protein S6 kinase*	Ovarian developmental stages and relative expression levels of *Vg*	2025	[22]
Xuewei Liu	*ribosomal protein L10a*	Relative expression levels of *Vg*	2024	[23]
Hongxia Jiang	*ribosome protein S24*	Ovarian developmental stages, relative expression levels of *Vg* and GSI	2024	[24]
Hongxia Jiang	*Cyclooxygenase*	Ovarian developmental stages, relative expression levels of *Vg* and GSI	2023	[25]
Hongxia Jiang	*ribosomal protein L24*	Ovarian developmental stages, relative expression levels of *Vg* and GSI	2022	[26]
Hongxia Jiang	*nm23*	Ovarian developmental stages, relative expression levels of *Vg* and GSI	2018	[27]

## Data Availability

The original contributions presented in this study are included in the article/Supplementary Materials. Further inquiries can be directed to the corresponding authors.

## Supplementary Materials

The following supporting information can be downloaded at: https://www.mdpi.com/article/10.3390/ijms262110748/s1.
