# Supplementary material for: Functional Analysis of Hyaluronidase-like Genes in Ovarian Development of Macrobrachium nipponense and Comparative Evaluation with Other Key Regulatory Genes
Source: Int J Mol Sci. 2025 Nov 5;26(21):10748. doi: 10.3390/ijms262110748 (PMC12608148; doi:10.3390/ijms262110748)
Supplement: Supplementary file 1 [file ijms-26-10748-s001.zip › Table S1.pdf]

Table S1. Specific classification criteria of ovary, hepatopancreas and embryonic development stages of *Macrobrachium nipponense*.

Stages	Characteristic
O1	oocyte stage, undeveloped stage, transparent, oocyte proliferation
O2	primary vitellogenesis stage, developing stage, yellow or khaki, original age of yolk
O3	secondary vitellogenesis stage, nearly-ripe stage, light green, secondary yolk
O4	ripe stage, dark green, yolk termination
O5	emptying stage, gray, recession
He1-He5	hepatopancreas corresponding to O1-O5 at the same stage
CS	cleavage stage, cell cleavage
BS	blastula stage, appearance of yolk sack
GS	gastrula stage, transparent area occurred on the end of embryo
NS	nauplius stage, formation of primordium
PS	protozoea stage, occurrence of eye pigments
ZS	zoea stage, formation of cephalothorax, appendage and interior structure
L1	1 day post hatching (larval stage)
L5	5 days post hatching (larval stage)
L10	10 days post hatching (larval stage)
L15	15 days post hatching (larval stage)
PL1	1 day post metamorphosis (postlarval stage)
PL5	5 days post metamorphosis (postlarval stage)
PL10	10 days post metamorphosis (postlarval stage)
PL15	15 days post metamorphosis (postlarval stage)
PL20	20 days post metamorphosis (postlarval stage)
PL25	25 days post metamorphosis (postlarval stage)
