# Supplementary material for: Functional Analysis of Hyaluronidase-like Genes in Ovarian Development of Macrobrachium nipponense and Comparative Evaluation with Other Key Regulatory Genes
Source: Int J Mol Sci. 2025 Nov 5;26(21):10748. doi: 10.3390/ijms262110748 (PMC12608148; doi:10.3390/ijms262110748)
Supplement: Supplementary file 1 [file ijms-26-10748-s001.zip › Table S2.pdf]

Table S2. Some tools and websites used in this study.

Function	Website or Tools	Access Time
ORF identification	https://www.ncbi.nlm.nih.gov/orffinder/	March 6, 2025
ORF verification primers and qPCR primers design	https://www.ncbi.nlm.nih.gov/tools/primer-blast/	March 6, 2025
dsRNA primers design	https://www.flyrnai.org/cgi-bin/RNAi_find_primers.pl	March 6, 2025
Protein physicochemical characteristics calculation	https://web.expasy.org/protparam/	March 7, 2025
Protein conserved domains prediction	https://www.ncbi.nlm.nih.gov/Structure/cdd/wrpsb.cgi	March 7, 2025
Protein functional domains and signal peptide prediction	https://www.ebi.ac.uk/interpro/	March 7, 2025
Transmembrane domains prediction	https://smart.embl.de/smart/set_mode.cgi?GENOMIC=1	March 7, 2025
Protein structures prediction	https://swissmodel.expasy.org/	March 8, 2025
Protein structure analysis	https://espript.ibcp.fr/ESPript/cgi-bin/ESPript.cgi	March 8, 2025
Amino Acid Sequence Alignment and Analysis	DNAMAN 9.0	March 9, 2025
Phylogenetic analysis	MEGA 11.0 (Neighbor-Joining Phylogenetic Tree Construction)	March 9, 2025
