# Supplementary material for: Functional Analysis of Hyaluronidase-like Genes in Ovarian Development of Macrobrachium nipponense and Comparative Evaluation with Other Key Regulatory Genes
Source: Int J Mol Sci. 2025 Nov 5;26(21):10748. doi: 10.3390/ijms262110748 (PMC12608148; doi:10.3390/ijms262110748)
Supplement: Supplementary file 1 [file ijms-26-10748-s001.zip › Table S3.pdf]

Table S3. The primers used in this study.

Primer	Primer Sequence
Mn-HyaL1 F1 (ORF)	TTCCAAGGCGATAAGGTGAACA
Mn-HyaL1 R1 (ORF)	AACTTCCCCATATGACAGAGCC
Mn-HyaL2 F1 (ORF)	ATCCGTAGATGGAGGTATCCCA
Mn-HyaL2 R1 (ORF)	AGAAATGACTTCGGCCATGAGT
Mn-HyaL1 F2 (qPCR, ORF)	AGCTGTATGCCGAGGCCAAGGA
Mn-HyaL1 R2 (qPCR, ORF)	TGTTCACCTTATCGCCTTGGAA
Mn-HyaL2 F2 (qPCR, ORF)	TTTCGCCCTAACCTTGTTAGCG
Mn-HyaL2 R2 (qPCR, ORF)	TGGGATACCTCCATCTACGGAT
EIF F (qPCR)	CATGGATGTACCTGTGGTGAAAC
EIF R (qPCR)	CTGTCAGCAGAAGGTCCTCATTA
dsMn-HyaL1 F (RNAi)	TAATACGACTCACTATAGGG
	GGTATCATCCGGGGAAGTTT
dsMn-HyaL1 R (RNAi)	TAATACGACTCACTATAGGG
	ATAGCCACAGGGTTTCGTTG
dsMn-HyaL2 F (RNAi)	TAATACGACTCACTATAGGG
	TACTGACTGGTTACACTACA
dsMn-HyaL2 R (RNAi)	TAATACGACTCACTATAGGG
	TCAGGATGCTGGGAGGATAC
dsMn-CH7D F (RNAi)	TAATACGACTCACTATAGGG
	CGCTAAAGTCCCGAAGACAG
dsMn-CH7D R (RNAi)	TAATACGACTCACTATAGGG
	ACGAATTTTGCGTAAGGTGC
dsMn-CTSL1 F (RNAi)	TAATACGACTCACTATAGGG
	GCTCTACAGGAAGACCATTTTC
dsMn-CTSL1 R (RNAi)	TAATACGACTCACTATAGGG
	GAAGTGAGCGAATTCCATTCC
dsMn-GIH F (RNAi)	TAATACGACTCACTATAGGG
	TCTCAACAAAGCTTTCACGC
dsMn-GIH R (RNAi)	TAATACGACTCACTATAGGG
	ACTTGCGTCCGACTCGTATT
dsGFP F (RNAi)	TAATACGACTCACTATAGGG
	ACGAAGACCTTGCTTCTGAAG
dsGFP R (RNAi)	TAATACGACTCACTATAGGG
	AAAGGGCAGATTGTGTGGAC
