# Supplementary figures and images for: Functional Analysis of Hyaluronidase-like Genes in Ovarian Development of Macrobrachium nipponense and Comparative Evaluation with Other Key Regulatory Genes
Source: Int J Mol Sci. 2025 Nov 5;26(21):10748. doi: 10.3390/ijms262110748 (PMC12608148; doi:10.3390/ijms262110748)

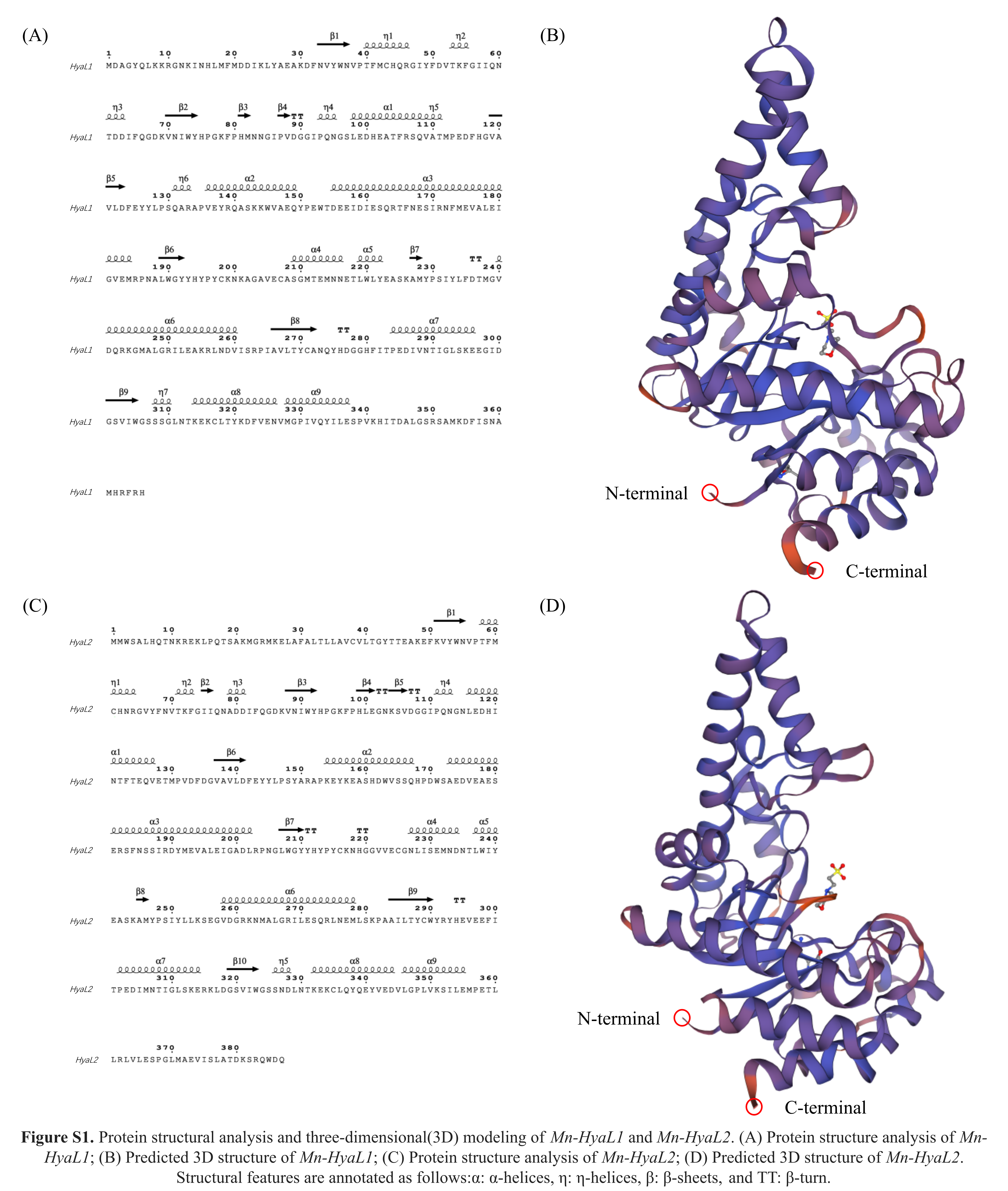

Supplement: Supplementary file 1 [file ijms-26-10748-s001.zip › Figure S1.tiff]

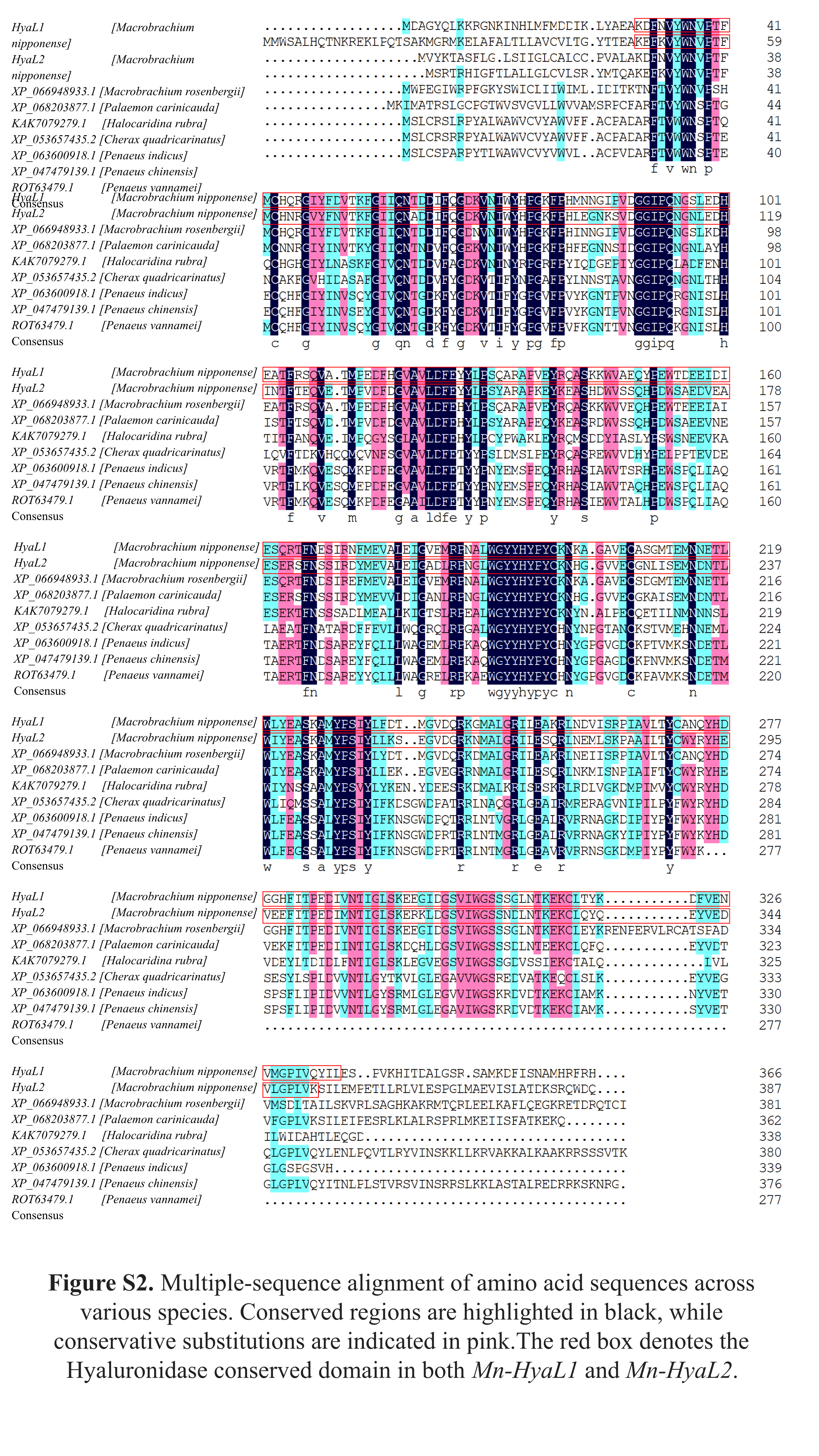

Supplement: Supplementary file 1 [file ijms-26-10748-s001.zip › Figure S2.tiff]
